# Homonymous Retinal Ganglion Cell Layer Atrophy With Asymptomatic Optic Tract Glioma in Neurofibromatosis Type I

**DOI:** 10.3389/fneur.2020.00256

**Published:** 2020-04-15

**Authors:** Amirah I. Momen, Ryan T. Muir, Carolina Barnett, Arun N. E. Sundaram

**Affiliations:** ^1^Division of Neurology, Department of Medicine, University of Toronto, Toronto, ON, Canada; ^2^Elizabeth Raab Neurofibromatosis Clinic, University Health Network, University of Toronto, Toronto, ON, Canada; ^3^Department of Ophthalmology and Vision Sciences, Sunnybrook Health Sciences Centre, University of Toronto, Toronto, ON, Canada

**Keywords:** neurofibromatosis, optic pathway glioma, optical coherence tomography, ganglion cell layer, optic tract, optic chiasm

## Abstract

Approximately 20% of patients with Neurofibromatosis type 1 (NF1) develop optic pathway gliomas (OPGs). Not all OPGs in NF1 necessarily become vision compromising and predicting which patients might develop visual decline is difficult at present time. Optical coherence tomography (OCT) has emerged as a useful tool able to directly assess the morphology and thickness of individual retinal layers. The ganglion cell layer (GCL) is composed of the retinal ganglion cells which receive information from photoreceptors via interneurons, while the retinal nerve fiber layer (RNFL) contains the retinal ganglion cell unmyelinated axons that merge to form the optic nerve. Lesions of the anterior visual pathway result in retrograde axonal degeneration from ganglion cell death and ultimately manifest as thinning of the RNFL and/or GCL. In this report we highlight a case of a 38 year-old woman with an NF1 associated left chiasmal and optic tract glioma who had normal visual fields and visual acuity. However, using OCT we demonstrate a homonymous pattern of GCL atrophy that corresponds with her left optic tract glioma. Given this homonymous pattern of atrophy in the GCL and the left optic tract lesion, one would expect a right homonymous hemianopia. To our knowledge this is the first reported case of a homonymous pattern of GCL-IPL atrophy in an adult with an NF1 related OPG involving the optic chiasm and optic tract, but without objective visual field or acuity deficits. This case is important because, mechanistically, it suggests that a necessary threshold of GCL atrophy may be needed before visual concerns can be detected and, secondly, it invites future studies to evaluate whether OCT may serve as a potential screening tool for those with NF1 related OPGs.

## Introduction

Neurofibromatosis type 1 (NF1) is an autosomal dominant neurocutaneous syndrome with a prevalence of 1 in 3,000 live births (1). Approximately 15–20% of patients with NF1 develop optic pathway gliomas (OPGs) (2). NF1 associated OPGs tend to be more indolent than sporadic forms with visual impairment occurring in less than half of cases (2). One study evaluated 414 consecutive patients with NF1 and 52 (15.4%) had an OPG (3). These patients were followed for an average of 11.9 years. In the asymptomatic OPG cohort 91.6% had normal visual outcomes over the study duration whereas 73.9% of the symptomatic OPG cohort had normal visual outcomes. Only 8.4% of the asymptomatic OPG cohort developed visual symptoms (3). At present time we have no way of knowing which asymptomatic patients with OPGs may become visually symptomatic and which visually symptomatic patients may have poor visual function. Others studies have not found any relationship between visual function and OPG location as visual outcomes are similar between optic nerve/chiasmal and retrochiasmal OPGs (4). While chemotherapeutic treatments are reserved for aggressive OPGs, surveillance remains a mainstay for mild and asymptomatic cases. In adults, however, there are no formal guidelines for the surveillance of OPGs and visual prognostication remains a challenge. However, Optical coherence tomography (OCT) could have an emerging role in the surveillance of OPGs in NF1.

OCT is a widely adopted, non-invasive imaging tool that allows for high resolution images of ocular tissues. OCT captures cross-sectional histological images with a resolution on the order of 10 micrometers, making it a useful modality for identifying abnormalities in the morphology and thickness of specific layers of the retina that are relevant to specific injuries or disease processes (5). While OCT has been used for over two decades in the study of ophthalmological disease, it is only in recent years that higher resolution OCT, known as spectral domain OCT, has allowed for segmentation and analysis of the various retinal layers. Our understanding of how quantitative measures of the individual retinal layers relates to both subclinical and clinical disease processes continues to evolve, but has especially been well-studied in the context of multiple sclerosis and optic neuritis. Three layers that are most commonly studied include the retinal nerve fiber layer (RNFL), ganglion cell layer (GCL), and inner plexiform layer (IPL). The GCL is composed of the retinal ganglion cells which receive information from photoreceptors via interneurons while the RNFL contains the retinal ganglion cell unmyelinated axons that merge to form the optic nerve. The portion of the RNFL immediately adjacent to the optic disc is known as the peripapillary retinal nerve fiber layer (pRNFL). The IPL is formed by the dendrites of the retinal ganglion cells and cells of the inner nuclear layer.

Lesions of the anterior visual pathway result in retrograde axonal degeneration from ganglion cell death and ultimately manifest as thinning of the RNFL. As a result of the IPL's close adherence to the GCL, the two are frequently measured in association and are referred to as the GCL-IPL. In recent years, studies have suggested that GCL-IPL thickness is more sensitive than RNFL or macular thickness in detecting clinically relevant changes in disease processes involving both the brain and optic nerve, such as multiple sclerosis (6, 7). As a result, there has been an increased interest in the measurement and comparison of the RNFL and GCL-IPL when assessing diseases of the neuro-ophthalmological axis.

In this case report we present a patient with NF1 and an asymptomatic OPG affecting the left optic chiasm and optic tract. Although she had a completely normal visual exam, optical coherence tomography (OCT) demonstrated atrophy of retinal ganglion cell layer and inner plexiform layer (GCL-IPL), but with preservation of peripapillary retinal nerve fiber layer (pRNFL) thickness. This case raises important questions regarding the pathophysiology of visual loss in OPGs and invites future investigations to examine the use of GCL-IPL OCT to assess asymptomatic optic pathway gliomas in the context of NF1. Our patient provided her informed consent for her case along with her MRI and OCT images to be published.

## Case

**Figure 1:**
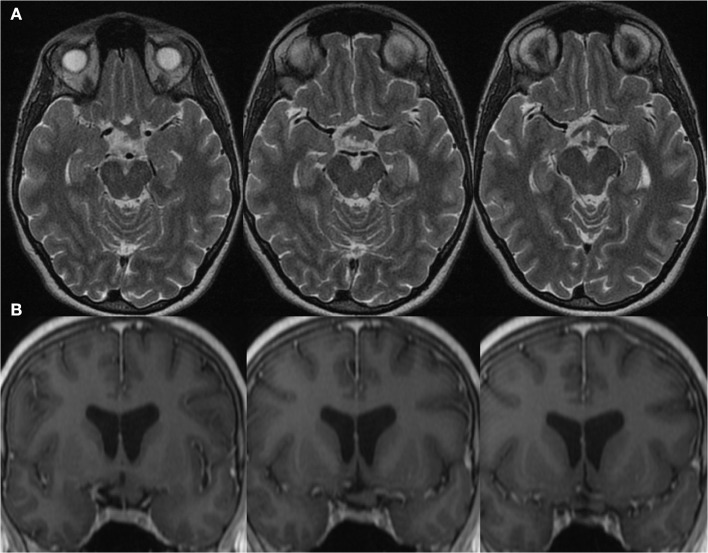
Magnetic Resonance Imaging of a left retro-chiasmal optic pathway glioma visualized on **(A)** Axial T2-MRI and **(B)** Coronal T1 post gadolinium MRI.

A 38 year-old woman with genetically confirmed Neurofibromatosis Type 1 (NF1) was assessed by Neuro-Ophthalmology for an asymptomatic left chiasmal and optic tract glioma newly discovered on screening Magnetic Resonance Imaging (MRI). This is depicted in Figure 1. It is uncertain for what duration she may have had this optic tract glioma as she was entirely asymptomatic from a visual perspective and this was the first time she underwent neuroimaging studies. She had other stigmata of NF-1 including, café-au-lait macules, axillary freckling, cutaneous neurofibromas and Lisch nodules. Her past medical history was otherwise unremarkable. She did not endorse any visual or neurological symptoms. On examination, her visual acuity was 20/20 bilaterally with no deficits noted on 24-2 Humphrey Visual Field analysis (mean deviation: + 0.18dB OD and −0.42dB OS) (see Supplementary Figures 1, 2). Pupils were equal and reactive to light with no relative afferent pupillary defect. Color vision was normal. Extraocular movements were full. Her optic disks appeared normal with healthy neuroretinal rims and no pallor. Her neurological examination was otherwise normal.

**Figure 2:**
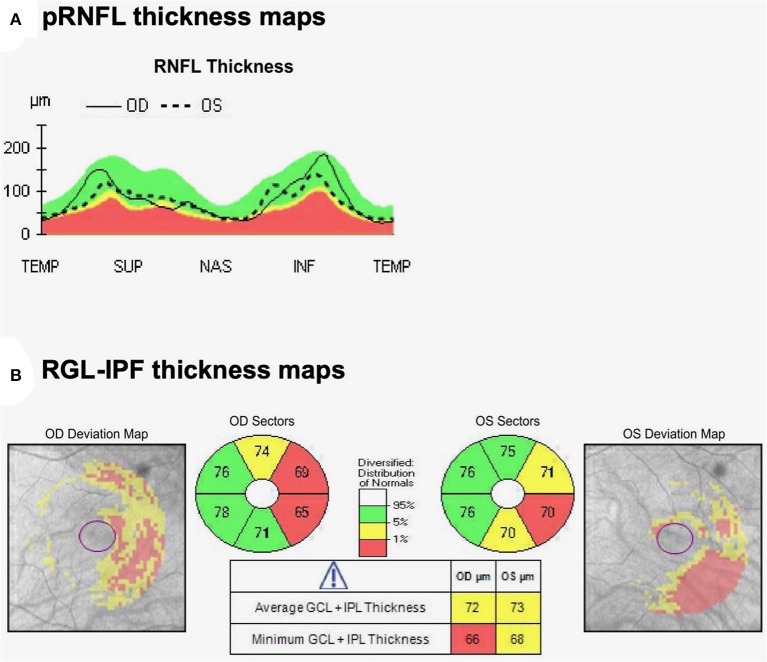
Optical coherence tomography **(A)** pRNFL and **(B)** Macular cube analysis by OCT demonstrates thinning of the nasal GCL-IPL OD and temporal GCL-IPL OS. Thicknesses reported are in micrometers. This represents a topographic pattern of GCL-IPL thinning that would correspond to a right homonymous visual field deficit, implicating a left optic tract localization.

OCT analysis revealed normal pRNFL thickness with average thickness 81 μm OD and 75 μm OS (Figure 2A). Most strikingly, however, despite preserved visual acuity and visual fields, OCT analysis of GCL-IPL thickness revealed a homonymous pattern of GCL-IPL atrophy (Figure 2B) reflecting loss of axons arising from the nasal retinal fibers of the right-eye and temporal retinal fibers of the left-eye. This pattern of atrophy would correspond to a right homonymous field deficit—the visual field deficit we would have expected our patient with a left optic tract glioma to have. As the patient was entirely asymptomatic from a visual standpoint, we suggested active annual surveillance of her visual fields and OCT metrics. No intervention was warranted at the time of consultation.

## Discussion

To the best of our knowledge there are no reports of adults with NF1 and asymptomatic OPGs with a normal exam and normal pRNFL demonstrating a topographic distribution of GCL-IPL atrophy that is consistent with the localization of the OPG. This is the first reported case of a homonymous pattern of GCL-IPL atrophy in an adult with an NF1 related OPG involving the optic chiasm and optic tract, without objective visual field or acuity deficits. Prior studies in patients with other optic pathway lesions have demonstrated GCL-IPL atrophy in those with normal visual function. For instance, a case series of patients with pituitary tumors but without a bitemporal hemianopsia had an expected pattern of bilateral nasal retinal GCL-IPL atrophy (8).

OCT as a surveillance tool in NF1 has almost exclusively been studied in children and has not been employed in adults. pRNLF thickness has been described as a sensitive and positive predictive measure of OPGs in children with NF1 (9) pRNFL atrophy has been described in a series of studies of children with NF1 related OPGs with visual deficits (10) and rarely in children with NF1 OPGs and no visual deficits (11). While pRNFL thickness is measured at the optic disc, OCT can simultaneously analyze the macular cubes to measure the GCL-IPL thickness. Gu et al. described GCL-IPL thinning in children with NF1 and OPGs (12). In that study, GCL-IPL was able to accurately discriminate between patients with normal and abnormal visual acuities (12). It is proposed that GCL-IPL is a more accurate and reliable biomarker of vision, because it is not confounded by axonal swelling, axonal atrophy, and blood vessel artifacts as in pRNFL analysis (12). In other studies, GCL-IPF thickness also correlates with visual acuity in children with OPGs (13). There is some evidence in children, therefore, that OCT may be a simple non-invasive surveillance tool for OPGs in NF1 as it objectively measures changes to pRNFL thickness that may result from damage to the anterior visual pathway. The evidence in adults is somewhat more limited. In adults with NF1 without OPGs or objective visual deficits, pRNFL and GCL-IPL thicknesses were decreased compared to controls (14). This raises the important possibility that smaller GCL-IPL thicknesses may also be mediated by NF1 itself, and not just dependent on the presence of an OPG. Whether GCL-IPF atrophy—in the context of normal visual acuity and fields—may predict future visual decline remains uncertain, and warrants further investigation. Given that our patient had an expected pattern of GCL-IPL atrophy that one would expect from a left optic tract lesion, this does beg the question of whether or not OCT could identify at risk cohorts of patients with NF-1 related OPGs. In order to answer this question, future cohort studies should prospectively follow patients with visually asymptomatic OPGs to ascertain whether GCL-IPF atrophy may indeed predict visual decline. There is some data to support that GCL-IPL atrophy may have prognostic value as it can precede visual decline in other optic neuropathies (8).

In our case, even though visual fields were preserved, the homonymous pattern of GCL-IPL atrophy corresponds to the expected visual field defect, a right homonymous hemianopia, given the patient's known location of her OPG in the left optic tract. The suspected pathophysiology for topographic GCL-IPL atrophy corresponding to an optic tract lesion is retrograde degeneration. Given our patient's preserved visual fields and acuity, our case does raise the scientific possibility that a threshold of GCL-IPL atrophy must first be met before visual problems manifest clinically. One might expect that with retrograde degeneration from the retinal ganglion cell axon toward the cell body there would be initial thinning of the RNFL with a normal GCL, however, our patient had a normal RNFL thickness with abnormal thinning of GCL-IPL. Studies of longitudinal RNFL and GCL-IPL changes in optic neuritis (ON) have suggested that this is a result of initial swelling of the axons within the RNFL, which does not occur within the GCL-IPL (6). It remains unclear whether an inflammatory cause of retrograde degeneration, such as ON, would have the same pathophysiology of retrograde degeneration due to an OPG.

OCT is currently used to monitor and inform treatment decisions in the context of idiopathic intracranial hypertension as it directly quantifies the severity of optic disc edema. We should therefore explore novel applications of OCT in other neuro-ophthalmologic conditions. However, OCT is not the only potential biomarker in NF1 related OPGs. Several other neuroimaging metrics are also being studied as biomarkers of visual function in OPG. For instance, the Diffusion Tensor Imaging (DTI) metric fractional anisotropy (FA) has been used to study the integrity of white matter tract optic radiations in NF1 associated OPGs. Even after adjusting for age, sex, extent of tumor, prior chemotherapy and fundus findings, decreased FA of the optic radiations was associated with abnormal visual acuity (2). Interestingly, initially decreased FA was predictive of visual decline over the subsequent year (2). Studying white matter tract integrity in this manner may be more sensitive to the microstructural damage caused by OPGs than conventional MRI alone (15). Nonetheless, MRI based DTI as a modality may not be widely available. OCT, in contrast, can be done in clinical settings and is a quick and inexpensive way to monitor and follow patients in clinic compared to MRI.

For patients living with NF-1, OPGs represent frightening, potentially vision compromising complications of their disease. While our case report cannot conclude that OCT is prognostic, it is the first report to suggest that, in asymptomatic NF1 related OPG, OCT may be a useful extension of the neurologic examination, by further identifying patterns of GCL-IPL atrophy that correspond to optic pathway lesions. As our report is based on a single case, these findings need further replication in large case series data. Other limitations of our report are: (i) it is unknown for how long this optic tract lesion had been present as the diagnosis of NF1 and the optic tract glioma were newly discovered and (ii) the absence of long-term follow up. This could have important implications on the axonal degeneration manifesting as GCL loss and on the detection of emerging visual field loss.

Overall, our findings in this report support a possible role for GCL-IPL analysis in the diagnosis and surveillance of NF1-related and asymptomatic OPGs. Future studies ought to prospectively follow visually asymptomatic patients with NF1 related OPGs to see whether the pattern of GCL-IPL atrophy may portend future visual decline. Our report raises the hypothesis that GCL-IPL atrophy may precede visual decline in OPGs and that there may be necessary threshold of GCL-IPL atrophy needed before visual concerns arise. Prospective studies utilizing OCT are needed to assess whether such a threshold exists and whether the rate of GCL-IPL atrophy over time may be predictive of future visual decline.

## Data Availability Statement

All datasets generated for this study are included in the article/Supplementary Material.

## Ethics Statement

The patient provided her written informed consent for her case along with her MRI and OCT images to be published.

## Author Contributions

AM and RM: study conception and design and manuscript preparation. CB: study conception and manuscript preparation. AS: study conception and design, manuscript preparation, acquisition of data, and final approval of manuscript.

### Conflict of Interest

The authors declare that the research was conducted in the absence of any commercial or financial relationships that could be construed as a potential conflict of interest.

## Supplementary Material

Supplementary Figures 1, 2 24-2 Humphrey Visual Field analysis revealing normal visual fields.
