# Supplementary figures and images for: Homonymous Retinal Ganglion Cell Layer Atrophy With Asymptomatic Optic Tract Glioma in Neurofibromatosis Type I
Source: Front Neurol. 2020 Apr 15;11:256. doi: 10.3389/fneur.2020.00256 (PMC7174762; doi:10.3389/fneur.2020.00256)

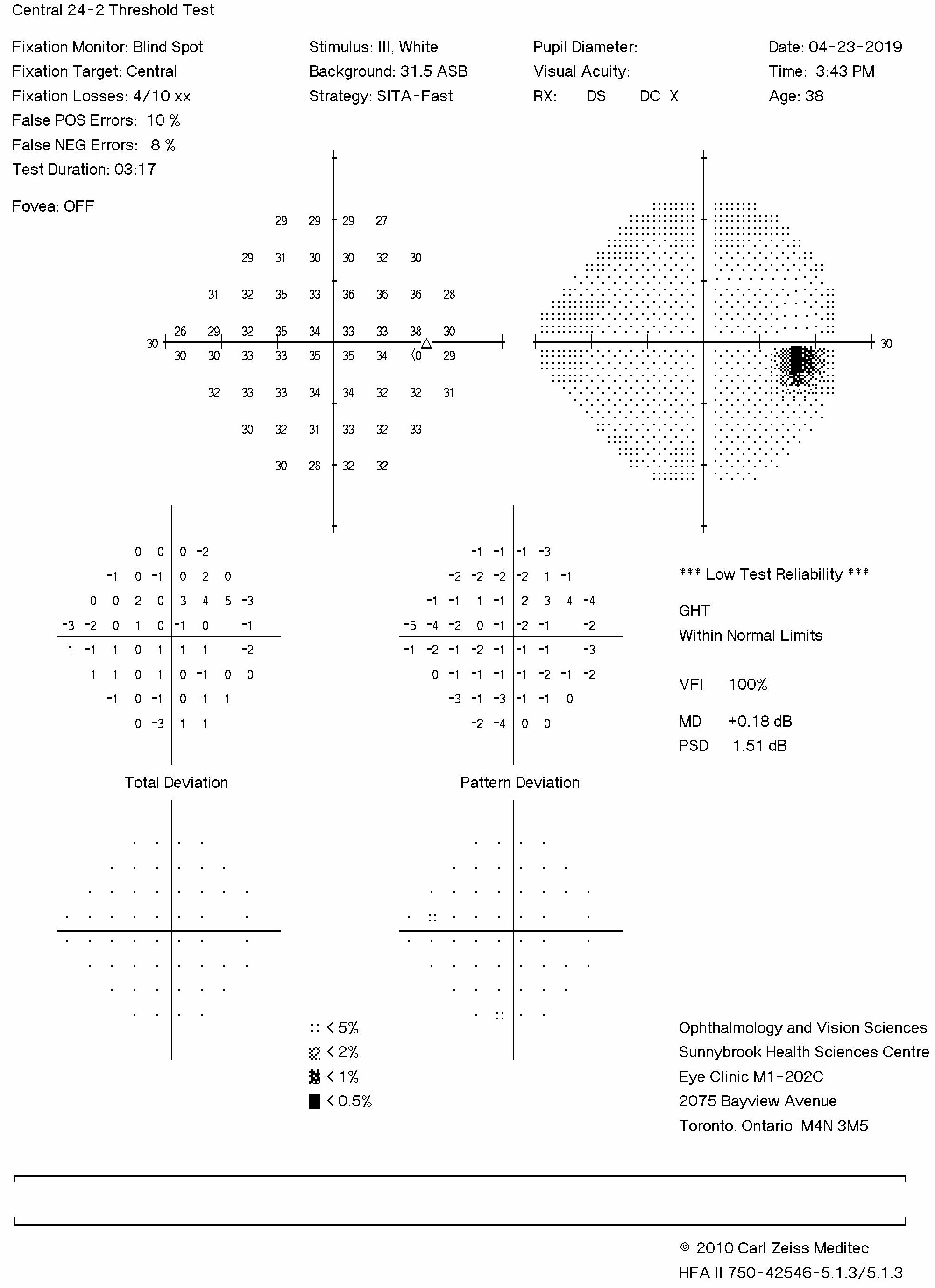

Supplement: Supplementary file 1 [file Image_1.JPEG]

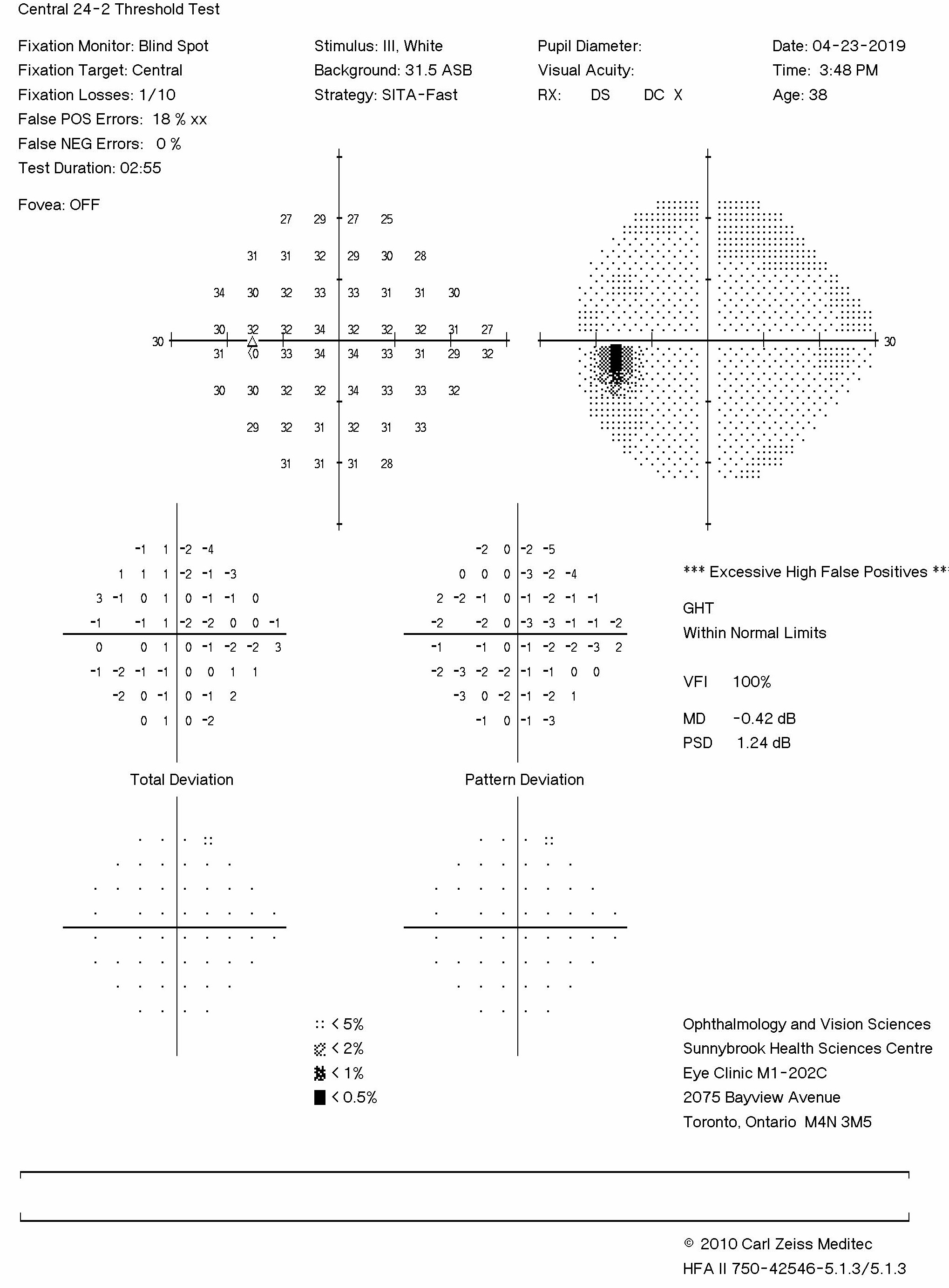

Supplement: Supplementary Figures 1, 2 — 24-2 Humphrey Visual Field analysis revealing normal visual fields. [file Image_2.JPEG]
